# Neuroprotective Effects of San-Huang-Xie-Xin-Tang in the MPP^+^/MPTP Models of Parkinson's Disease *In Vitro* and *In Vivo*

**DOI:** 10.1155/2012/501032

**Published:** 2012-03-05

**Authors:** Yi-Ching Lo, Yu-Tzu Shih, Yu-Ting Tseng, Hung-Te Hsu

**Affiliations:** ^1^Department of Pharmacology, School of Medicine, College of Medicine, Kaohsiung Medical University, 100 Shih-Chuan 1st Road, Kaohsiung 80708, Taiwan; ^2^Graduate Institute of Natural Products, Kaohsiung Medical University, Kaohsiung 80708, Taiwan; ^3^Graduate Institute of Medicine, Kaohsiung Medical University, Kaohsiung 80708, Taiwan; ^4^Department of Anesthesia, Kaohsiung Medical University Hospital, Kaohsiung 80708, Taiwan

## Abstract

San-Huang-Xie-Xin-Tang (SHXT), composed of *Coptidis rhizoma, Scutellariae radix*, and *Rhei rhizoma*, is a traditional Chinese medicine used for complementary and alternative therapy of cardiovascular and neurodegenerative diseases via its anti-inflammatory and antioxidative effects. The aim of this study is to investigate the protective effects of SHXT in the 1–methyl–4–phenylpyridinium (MPP^+^)/1–methyl–4–phenyl–1,2,3,6–tetrahydropyridine (MPTP) models of Parkinson's disease. Rat primary mesencephalic neurons and mouse Parkinson disease model were used in this study. Oxidative stress was induced by MPP^+^ *in vitro* and MPTP *in vivo*. In MPP^+^-treated mesencephalic neuron cultures, SHXT significantly increased the numbers of TH-positive neurons. SHXT reduced apoptotic signals (cytochrome and caspase) and apoptotic death. MPP^+^-induced gp91^phox^ activation and ROS production were attenuated by SHXT. In addition, SHXT increased the levels of GSH and SOD in MPP^+^-treated neurons. In MPTP animal model, SHXT markedly increased TH-positive neurons in the substantia nigra pars compacta (SNpc) and improved motor activity of mice. In conclusion, the present results reveal the evidence that SHXT possesses beneficial protection against MPTP-induced neurotoxicity in this model of Parkinson's disease via its antioxidative and antiapoptotic effects. SHXT might be a potentially alternative and complementary medicine for neuroprotection.

## 1. Introduction

San-Huang-Xie-Xin-Tang (SHXT) is a traditional Chinese medicinal formula, containing *Coptidis rhizoma *(*Coptis chinesis *Franch), *Scutellariae radix *(*Scutellaria baicalensis *Georgi), and *Rhei rhizoma *(*Rheum officinale *Baill). SHXT is traditionally used to treat various diseases via its anti-inflammatory effects. Some of the major components (e.g., baicalin, baicalein, wogonin, and emodin) in SHXT have identified their potential protection on neuron. Baicalin protects against cerebral ischemia/reperfusion *in vivo* and oxygen-glucose deprivation *in vitro* [1, 2]. Baicalein prevents 6-OHDA-induced experimental parkinsonism [3], ischemia injury [4], and traumatic brain injury [5]. Baicalein [6] and wogonin [7] attenuate inflammation-mediated neurodegeneration. Emodin, an anthraquinone derivative extracted from *Rhei rhizoma*, decreased glutamate excitotoxicity [8] and *β*-amyloid-induced neurotoxicity [9]. Accordingly, SHXT is standardized and pharmacologically investigated in our lab. We demonstrate that SHXT attenuates lipopolysaccharide (LPS) and *Helicobacter pylori*-induced inflammatory responses [10–12]. We also reveal the potential benefits of SHXT used in the treatment of pulmonary hypertension [13]. We further prove the potential protective mechanisms of SHXT on activated microglia- and 6-OHDA-induced neurotoxicity via its anti-inflammatory and antioxidative properties [14].

Oxidative stress has been implicated in the progression and cellular damage in many neurodegenerative disorders, such as Parkinson's disease (PD), Alzheimer's disease (AD), and amyotrophic lateral sclerosis (ALS) [15]. PD is a progressive neurodegenerative disease characterized by a loss of dopaminergic neurons in substantia nigra pars compacta (SNpc) and can be modeled by the neurotoxin 1-methyl-4-phenyl-1,2,3,6-tetrahydropyridine (MPTP) [16]. Oxidative stress-induced mitochondrial dysfunction is recognized as a key player in the pathogenesis of PD [17]. MPTP can cross the blood-brain barrier and converts to its active metabolite 1-methyl-4-phenylpyridinium (MPP^+^) [18], which is selectively accumulated by high-affinity dopamine transporters into the mitochondria of dopaminergic neurons and induced dopaminergic neuron death [19]. Since SHXT possesses novel anti-inflammatory and antioxidative effects, therefore, we aimed to investigate the protective effects and mechanisms of SHXT on oxidative stress-induced neurotoxicity by using MPP^+^ *in vitro* model and MPTP mouse model of Parkinson's disease.

## 2. Materials and Methods

### 2.1. Materials

The blended mixture of *Coptidis rhizoma*, root of *Scutellariae radix,* and *Rhei rhizoma* was prepared in a ratio of 1 : 1 : 2, respectively. The voucher specimen, method for extraction and contents of each component in SHXT were described in our previous study [11]. Briefly, the mixture was extracted with 5 parts distilled water for 1 h, centrifuged at 1500 ×g at room temperature to obtain the supernatant and then concentrated under reduced pressure at 65°C to obtain the semisolid form of SHXT (yield: 23.2%), which was made up by distilled water to contain 60% water. The content (*μ*g/mL) of each component in SHXT analyzed by HPLC is as follows: baicalin 1153.07 ± 56.36, baicalein 82.81 ± 3.74, emodin 11.15 ± 1.22, wogonin 19.55 ± 0.83, rhein 126.12 ± 3.84, berberine 62.14 ± 4.27, coptisine 6.15 ± 0.34, palmatine 25.11 ± 3.78, sennoside A 128.02 ± 13.56, and sennoside B 95.90 ± 3.59. Minimum essential medium (MEM), fetal bovine serum (FBS), horse serum, glutamine, B27, nonessential aminoacids, sodium pyruvate, penicillin, amphotericin B, streptomycin, and Alexa Fluor 488 goat anti-rabbit IgG (H + L) were obtained from Invitrogen (Carlsbad, CA, USA). All materials for SDS-PAGE were obtained from Bio-Rad (Hercules, CA, USA). Mouse antibodies against cytochrome *c*, rabbit antibody against TH and caspase-3, and all horseradish peroxidase-conjugated secondary antibodies were obtained from Santa Cruz Biotechnology (Santa Cruz, CA, USA). Mouse antibody against gp91^phox^ was obtained from BD Bioscience (San Jose, CA, USA). Mouse anticaspase-9 was obtained from Millipore (Bedford, MA, USA). Enhanced chemiluminescence reagent and polyvinylidene difluoride (PVDF) membrane were purchased from PerkinElmer Life and Analytical Sciences (Boston, MA, USA). LDH cytotoxicity assay kit was purchased from G-Biosciences (St. Louis, MO, USA). Annexin-V-FITC assay kit was obtained from Strong Biotech Corporation (Taipei, Taiwan). Glutathione measurement kit and superoxide dismutase activity kit were purchased from Assay Designs (Ann Arbor, MI, USA). Simple stain mouse MAX PO was purchased from Nichirei Biosciences (Tokyo, Japan). Diaminobenzidine (DAB) kit was obtained from BioGenex (San Ramon, CA, USA). All other materials were purchased from Sigma Chemical Company (St. Louis, MO, USA).

### 2.2. Animals

All animals used for this study were approved by the Animal Care and Use Committee at the Kaohsiung Medical University. Pregnant Sprague-Dawley rats and male C57BL/6 mice (7-8 weeks old, 20–25 g) were purchased from the National Laboratory Animal Breeding and Research Center (Taipei, Taiwan). They were housed under conditions of constant temperature and controlled illumination (lights on between 7 : 30 and 19:30).

### 2.3. MPTP Mouse Model

 Experimental Parkinson's disease model was established by intraperitoneal (*i.p.*) injection of MPTP (20 mg/kg four times at 2 h interval). Mice received SHXT (10 mg/kg or 20 mg/kg, *i.p.*, once per day) for 7 days while MPTP was given on the 8th day. Control animals received saline only. Mice have to undergo locomotor activity assay 7 days after MPTP treatment and sacrificed for TH immunohistochemistry.

### 2.4. Primary Mesencephalic Neuron Cultures

Primary mesencephalic neuron cultures were prepared from gestation day 15-16 rat embryos (E15-16). In brief, meninges-free ventral mesencephalon were isolated and suspended. Cells were plated onto 6-, 24-, or 96- well plates precoated with 20 *μ*g/mL poly-L-lysine in MEM containing 10% FBS, 10% horse serum, 1 g/l glucose, 2 mM L-glutamine, 1 mM sodium pyruvate, 100 *μ*M nonessential aminoacids, 100 U/mL penicillin, 100 *μ*g/mL streptomycin, and 0.25 *μ*g/mL amphotericin B at 37°C in a humidified incubator under 5% CO_2_ and 95% air. After 24 h incubation, culture medium was replaced by MEM supplemented with 2% B27 and 10 *μ*M cytosine arabinoside to control glia proliferation. After further 48 h, the medium was replaced with MEM containing 2% B27 and the cells were allowed to develop *in vitro* for 6 days.

### 2.5. MTT Assay

Cell viability was measured by a quantitative colorimetric assay with MTT, showing the mitochondrial activity of living cells. After indicated treatments, cells were incubated with 0.1 mg/mL MTT for 3 h at 37°C. The reaction was determined by addition of 100 *μ*L DMSO. The amount of MTT formazan product was determined by measuring the absorbance at 560 nm using a microplate reader.

### 2.6. LDH Assay

Cytotoxicity was evaluated by colorimetric assay based on the measurement of lactate dehydrogenase (LDH) activity release from damaged cells into the supernatant. The release of LDH is measured with a coupled enzymatic reaction using a cytotoxicity detection kit that results in the conversion of a tetrazolium salt into a red color formazan. The amount of formazan formed correlated with LDH activity. The formazan product was measured with a microplate reader at 490 nm.

### 2.7. TH Immunocytochemistry

Immunocytochemistry assay was used to detect the expression of tyrosine hydroxylase (TH) and used to estimate the number of dopaminergic neurons in neuron cultures. After the cells were pretreated with SHXT for 1 h in 24-well plates and followed by exposure to MPP^+^ for 48 h, cells were fixed with 4% paraformaldehyde for 30 min. Then, cells were incubated in 0.2% Triton X-100 for 10 min, twice wash with PBS, and incubated with 2% BSA for 1 h. The cells were then incubated with the anti-TH primary antibody (1 : 3000) overnight at 4°C. Alexa Fluor 488 goat anti-rabbit IgG (H + L) was used as the secondary antibody. Viable neurons were then enumerated under a fluorescence microscope. Six separate cultures were analyzed for each treatment and five representative fields were counted per culture.

### 2.8. Apoptosis Detection

Apoptotic neuronal cells were detected by the use of double staining with Annexin V-FITC/PI according to the manufacturer's instructions. Briefly, cells were detached from plastic dishes and washed twice with cold PBS. The cell pellets were suspended in 1× binding buffer (10 mM HEPES/NaOH, pH7.4, 140 mM NaCl, 2.5 mM CaCl_2_) at a concentration of 1 × 10^6^ cells/mL. Then the cells were incubated with AnnexinV-FITC and propidium iodide (PI) for 15 min (22–25°C) in dark. The stained cells were immediately analyzed by flow cytometry (Partec, Germany).

### 2.9. Determination of Reactive Oxygen Species

The level of ROS was quantified by fluorescence with H_2_DCF-DA. Following incubations with the indicated treatments, neurons were loaded with 10 *μ*M of H_2_DCF-DA for 20 min at 37°C. Cells were detached from plates and washed with PBS. 10^5^ cells were analyzed by a Coulter CyFlow Cytometer (Patrec, Germany). DCF fluorescence was measured using an excitation of 495 nm and emission of 520 nm.

### 2.10. Glutathione Quantification

Glutathione (GSH) measurement kit was purchased from Assay Designs (USA). Neurons were grown on 12-well plates for 6 days. After indicated treatment, neurons were then collected and washed with PBS. After centrifuge, the pellets were suspended and deproteinated in 5% metaphosphoric acid by brief sonication. After centrifugation at 13,000 ×g for 5 min, collect the supernatants for total glutathione measurement. GSH was determined by adding reaction Mix and GSH reductase supplied in the kit, following incubation and measurement by ELISA reader at 405 nm for 20 min at 1 min interval. The total amount of GSH was determined by means of a calibration curve and normalized to the protein concentration, which was quantified by Bio-Rad protein assay kit.

### 2.11. Superoxide Dismutase Activity Assay

Superoxide dismutase (SOD) activity kit was purchased from Assay Designs (USA). This method was assayed by xanthine oxidase and conversion of WST-1 to WST-1 formazan. Neurons were grown on 12-well plates for 6 days. After indicated treatment, neurons were then harvested and cytosolic protein was extracted. SOD activity was determined by adding Master Mix and xanthine supplied in the kit, following incubation and measurement by ELISA reader at 450 nm for 10 min at 1 min interval. Protein concentration was quantified by Bio-Rad protein assay kit. Then, calculate SOD activity in cell extracts versus SOD standard curve. 

### 2.12. Western Blotting Analysis

After indicated treatment, neurons were collected and lysed to determine the expression of cytochrome *c*, caspase-9, caspase-3 and gp91^phox^. For the detection of cytochrome *c* release, the cells were fractionated using a mitochondria/cytosol fractionation kit according to the manufacturer's instruction (BioVision, USA). Protein concentration was determined with the Bio-Rad protein assay kit following the manufacturer's guide. Equal amounts of protein were separated by a polyacrylamide gel and transferred to PVDF membranes. Nonspecific binding was blocked with TBS-T (50 mM Tris-HCl, pH 7.6, 150 mM NaCl, 0.1% Tween 20) containing 5% nonfat milk for 1 h at room temperature. The membranes were then incubated overnight at 4°C with one of the following specific primary antibodies: mouse anti-cytochrome *c* (1 : 500), mouse anti-caspase-9 (1 : 1000), rabbit anti-caspase-3 (1 : 200), mouse anti-gp91^phox^ (1 : 500) and mouse anti-*β*-actin (1 : 20000). Membranes were washed six times 5 min each with TBS-T. The appropriate dilutions of secondary antibodies were incubated for 1 h. Following six washes with TBS-T, protein bands were detected with ECL reagent. Protein blot images were captured by an Imaging Densitometer with the aid of software (Bio-ID, V.97 software for Windows 95, Vilber Laurmat, France). Comparisons were made only between average values of bands within the same gel.

### 2.13. Behavior Analysis of Locomotor Activity

Locomotor activity was assessed in chambers (50 cm × 50 cm × 25 cm) connected to a digiscan analyzer that transmitted the number of beam breaks (activity data) to a computer. The locomotor activity was recorded as total distance (cm) and mean velocity (cm/s) in the 10 min recording period.

### 2.14. TH Immunohistochemistry

TH immunohistochemistry has been widely used as an important method of detecting the injury or death of dopaminergic neurons [20, 21]. All animals were anesthetized with chloral hydrate and the brains were perfusion fixed with 4% paraformaldehyde. The brains were then dissected and postfixed in 4% paraformaldehyde overnight at 4°C. Then, the tissues were dehydrated and embedded in paraffin wax. 5 *μ*m thick coronal sections were then cut through the ventral mesencephalon. Sections were stained with rabbit antibody against TH (1 : 100) for 1 h at room temperature. After PBS wash, sections were incubated in simple stain mouse MAX PO by the Universal Immunoenzyme Polymer (UIP) method for 30 min. The sections were then incubated in DAB substrate for 10 min and then dehydrated in graded series of alcohol and xylene. TH-positive neurons were counted in six sections of the sunstantia nigra region.

### 2.15. Statistical Analysis

Data were expressed as mean ± S.E.M. Analysis of variance (ANOVA) was used to assess the statistical significance of the differences followed by Dunnett's test for comparison of multiple means. Probability values (*p*) less than 0.05 were considered to be significant in all experiments. All data were analyzed with the Statistical Package for the Social Sciences (SPSS, Chicago, IL) program version 14.0.

## 3. Results

### 3.1. SHXT Attenuates MPP^+^-Induced Cytotoxicity in Rat Primary Mesencephalic Neurons

**Figure 1:**
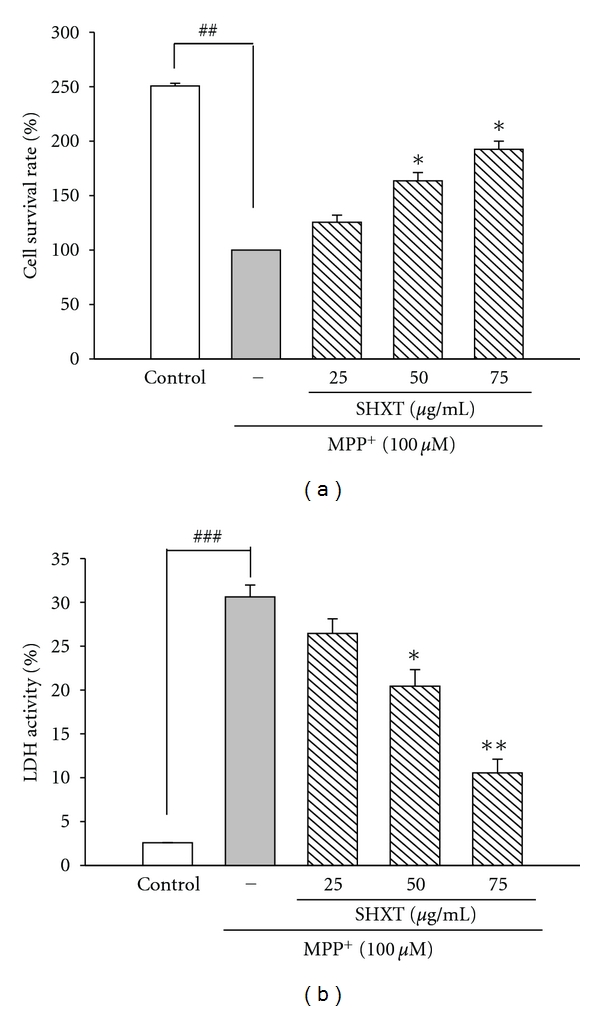
Effects of SHXT on MPP^+^-induced cell death of primary mesencephalic neurons. Cells were treated with SHXT (25–75 *μ*g/mL) 1 h prior to MPP^+^ (100 *μ*M) addition for 48 h. (a) Cell viability was determined by MTT assay. (b) Cytotoxicity was measured by LDH assay. Bars represent the mean ± S.E.M. from six independent experiments. ^##^ *P* < 0.01, ^###^ *P* < 0.001 versus control (without any treatment), *P < 0.05, **P < 0.01 versus MPP^+^ only.

**Figure 2:**
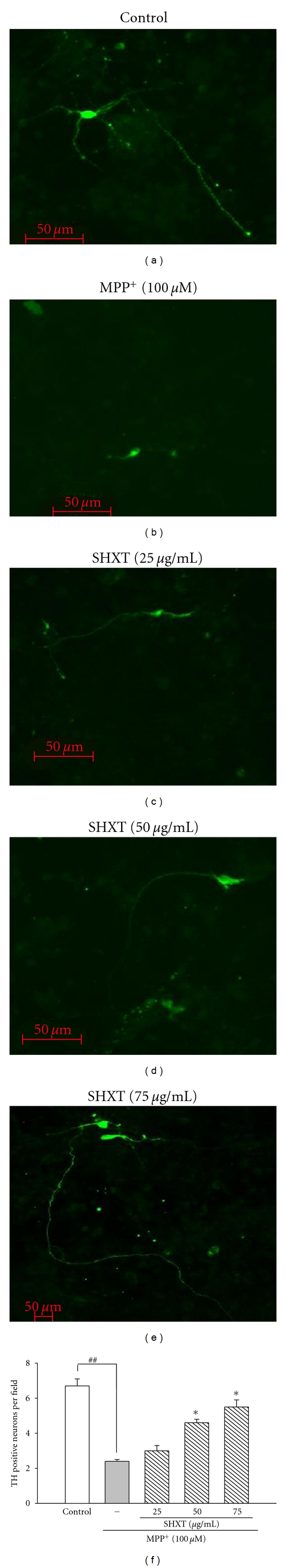
Effects of SHXT on MPP^+^-induced changes of TH staining (a)–(e) and numbers of TH-positive neurons (f) in rat primary mesencephalic neurons. Primary mesencephalic neurons ((a), control) treated with SHXT (25–75 *μ*g/mL) 1 h prior to MPP^+^ (100 *μ*M) addition for 48 h were further confirmed by staining with anti-TH antibody (green). Numbers of TH-positive neurons were counted under a fluorescent microscope. Bars represent the mean ± S.E.M. from six independent experiments. ^##^ *P* < 0.01 versus control (without any treatment), *P < 0.05 versus MPP^+^ only. Scale bar = 50 *μ*m.

In order to evaluate the effect of SHXT on MPP^+^-induced toxicity, mesencephalic neurons were treated with SHXT (25–75 *μ*g/mL) 1 h prior to MPP^+^ (100 *μ*M) addition for 48 h [22]. Results from MTT test and LDH assay indicated MPP^+^ significantly induced neuronal cell death (Figures 1(a) and 1(b)). However, SHXT showed a neuroprotective effect against MPP^+^-induced cytotoxicity in rat primary mesencephalic neurons. We further identified the effect of SHXT on dopaminergic neuron phenotype by tyrosine hydroxylase (TH) immunocytochemistry. Results that indicated the processes of dopaminergic neuron in MPP^+^-treated cultures (Figure 2(b)) were shorter or completely absent and the number of TH-positive neurons was significantly lower than that observed in the control (without any treatment) (Figure 2(a)). Pretreatment of SHXT can reduce MPP^+^-induced loss and death of dopaminergic neurons (Figures 2(c)–2(f)).

### 3.2. SHXT Attenuates MPP^+^-Induced Oxidative Stress by Decreasing ROS Production and gp91^phox^ Activation and Enhanced GSH Level and SOD Activity

**Figure 3:**
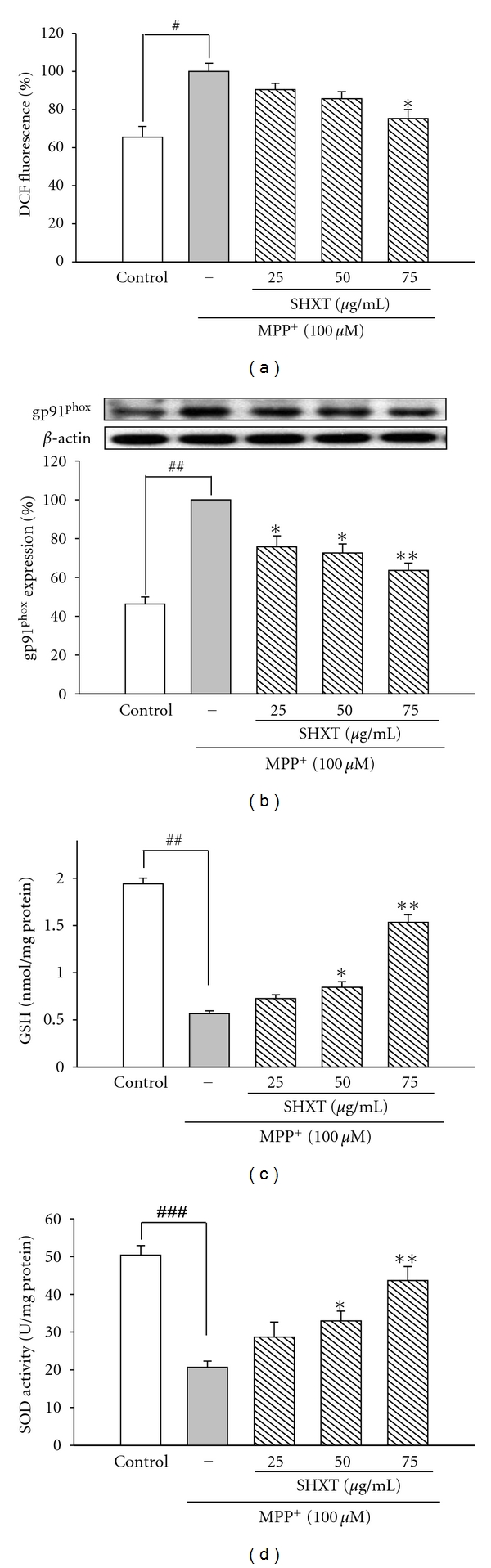
Effects of SHXTon ROS level (a), gp91^phox^ expression (b), GSH level (c), and SOD activity (d) in primary mesencephalic neurons treated with MPP^+^ for 48 h. Cultures were pretreated with SHXT (25–75 *μ*g/mL) for 1 h before MPP^+^ treatment. ROS was determined by H_2_DCF-DA staining. Protein expression was detected by western blotting. GSH level and SOD activity were measured by commercial kits. Bars represent the mean ± S.E.M. from six independent experiments. Densitometry analyses are presented as the relative ratio of protein/*β*-actin protein and are represented as percentages of MPP^+^ group. ^#^ *P* < 0.05, ^##^ *P* < 0.01 versus control (without any treatment), *P < 0.05, **P < 0.01 versus MPP^+^ only.

As the oxidative stress is the main cause of MPP^+^-induced cytotoxicity, we investigated the effect of SHXT on MPP^+^-induced ROS generation by measuring H_2_DCF-DA loaded neuronal cells using flow cytometry. Results indicated MPP^+^-induced increase in ROS production was attenuated by SHXT pretreatment (Figure 3(a)). Moreover, MPP^+^-induced gp91^phox^ overexpression, which plays an important role in ROS production, was also attenuated by SHXT pretreatment (Figure 3(b)). Furthermore, MPP^+^ significantly decreased GSH level and SOD activity in the rat primary neurons. However, SHXT could significantly increase GSH level (Figure 3(c)) and SOD activity (Figure 3(d)) compared with MPP^+^-treated group.

### 3.3. SHXT Decreased MPP^+^-Induced Apoptotic Signaling and Death

**Figure 4:**
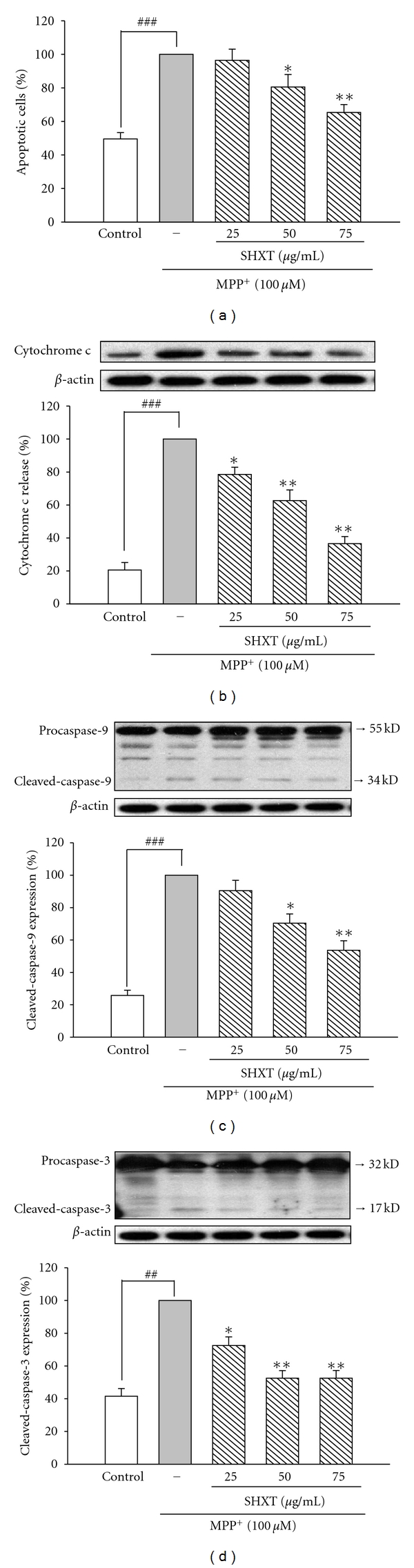
SHXT decreased numbers of apoptotic cells (a), cytochrome *c* release (b), cleavage of caspase-9 (c), and caspase-3 (d) in primary mesencephalic neurons treated with MPP^+^(100 *μ*M) for 48 h. SHXT (25–75 *μ*g/mL) were treated to neurons 1 h prior to MPP^+^ addition. Apoptotic cells were stained with Annexin V and PI after MPP^+^ exposure for 48 h. Apoptosis degree of each group was shown as apoptosis index evaluated by counting the percentage of apoptotic cells (Annexin V-positive cells) using flow cytometry. Protein expression was detected by western blotting. Densitometry analyses are presented as the relative ratio of protein/*β*-actin protein. Data are represented as percentage of MPP^+^ group from six independent experiments. ^###^ *P* < 0.05 versus control (without any treatment), *P < 0.05, **P < 0.01 versus MPP^+^ only.

As MPP^+^-induced neurotoxicity has been linked to apoptosis, we assessed the effect of SHXT on MPP^+^-induced apoptosis of primary mesencephalic neurons by flow cytometry analysis using Annexin V/PI staining. Results showed MPP^+^-induced apoptosis could be attenuated by SHXT treatment (Figure 4(a)). We further evaluated the effects of MPP^+^-induced apoptosis-related proteins. In MPP^+^-treated neurons, SHXT decreased the release of cytochrome *c* from mitochondria to cytosol (Figure 4(b)) and the cleavage of procaspase-9 and procaspase-3 (Figures 4(c) and 4(d)).

### 3.4. SHXT Attenuated MPTP-Induced Loss of TH-Positive Neurons and Improved Locomotor Activity in MPTP-Treated Mice

**Figure 5:**
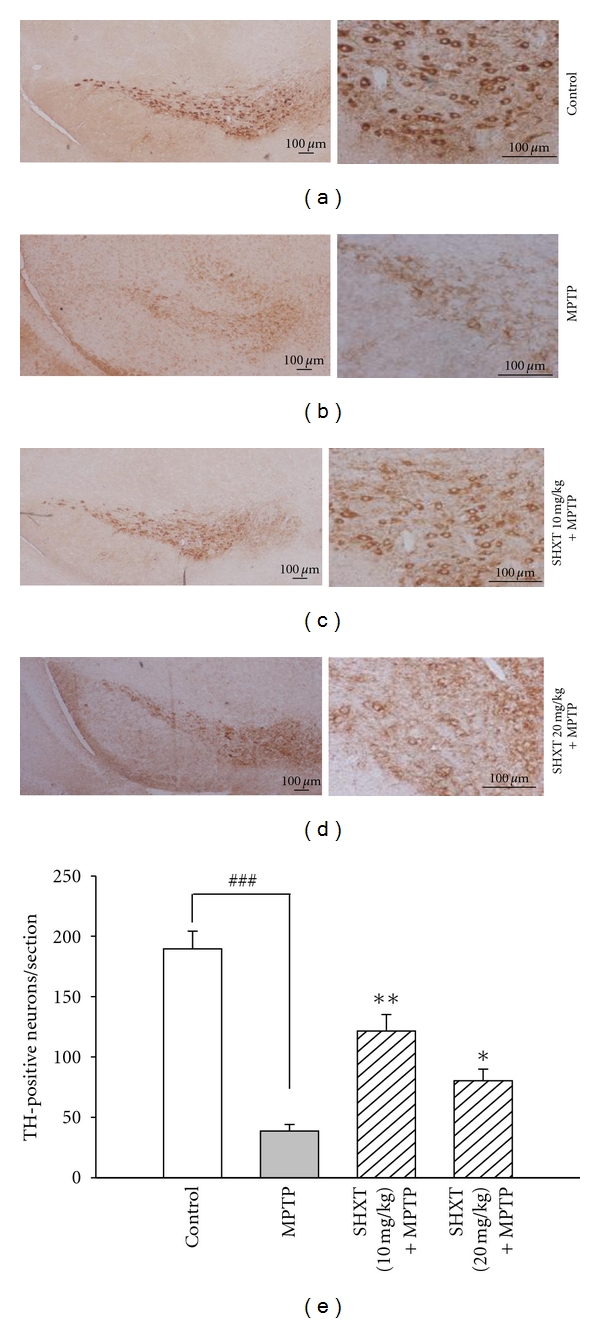
SHXT increased TH-positive neurons in the substantia nigra par compacta (SNpc) of MPTP-treated mice. C57BL/6 mice were treated with SHXT (10 mg/kg or 20 mg/kg, *i.p.*) for 7 days and then MPTP (20 mg/kg, 4 times, 2 h interval, *i.p.*) was injected on 8th day. Mice were sacrificed on the 7th day after MPTP injection. (a): Control group, (b): MPTP group, (c): SHXT (10 mg/kg) + MPTP, and (d): SHXT (20 mg/kg) + MPTP. Scale bar = 100 *μ*m. (e): Data represented as mean ± S.E.M. from six independent experiments. ^###^ *P* < 0.001 versus control (saline only), *P < 0.05, **P < 0.01 versus MPTP group.

**Figure 6:**
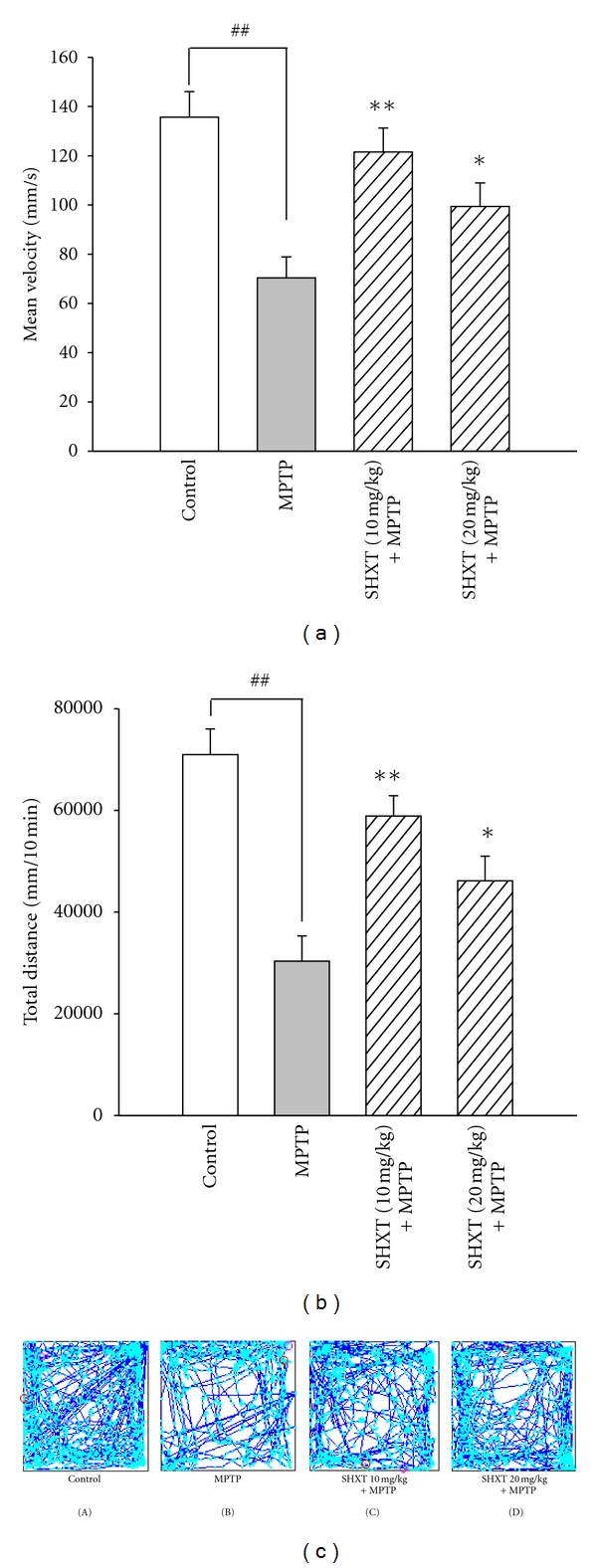
Effects of SHXT on the locomotor activity in MPTP-treated mice. Locomotor activity was detected in 10 min. Data of mean velocity (a) and total movement (b) were shown as mean ± S.E.M. from six independent experiments. ^##^ *P* < 0.01 versus control (saline only), *P < 0.05, **P < 0.01 versus MPTP group. (c) shows the effects of SHXT on the 10-mintues track plot pictures MPTP mice. (A), control; (B), MPTP; (C), SHXT (10 mg/kg) + MPTP; (D), SHXT (20 mg/kg) + MPTP.

As shown in Figure 5, MPTP exposure leads to a markedly loss of TH-positive neurons in the SNpc compared to the control group. However, SHXT pretreatment significantly reduced the loss of TH-positive neurons. Furthermore, in comparison with the control group, the MPTP-treated mice displayed a significant decrease in locomotor activity by measuring mean velocity and total movement distance (Figures 6(a) and 6(b)), which were reversed by SHXT treatment.  Figure 6(c) showed the effects of SHXT on the 10 min track plot pictures of MPTP mice.

## 4. Discussion

 PD is the second most prevalent age-related neurodegenerative diseases, primarily affecting people of ages over 55 years with physiological manifestations [23]. In the present study, we provide the evidence that the traditional Chinese medicine SHXT possesses novel protective effects in the MPP^+^/MPTP models of PD. *In vitro* study indicates SHXT protects dopaminergic neurons from the damage induced by MPP^+^. SHXT attenuates MPP^+^-induced oxidative stress by decreasing ROS production and gp91^phox^ expression. SHXT also enhances antioxidative defense by increasing GSH level and SOD activity. SHXT also decreases apoptosis-related signal and apoptotic death induced by MPP^+^. *In vivo* study shows SHXT treatment significantly increased TH-positive neurons in the SNpc and improved motor activity in MPTP mice.

 The antioxidant and anti-inflammatory effects of SHXT have been demonstrated in experimental models of various diseases [10–14]. Oxidative damage may occur in the parkinsonian brain and is responsible for neurodegeneration. Activation of NADPH oxidase is regarded as a major source of superoxide in a number of neurodegenerative diseases, including PD. The neurotoxicity of MPTP was diminished in mice lacking functional NADPH oxidase [24]. Furthermore, antioxidant status is contributive to the protection of MPTP-induced dopaminergic neurons loss. For instance, MPTP depletes striatal GSH in mice, and this effect may make dopaminergic neurons more susceptible to oxidative stress [25]. SOD is the most important enzyme for the detoxification of ROS and protection against oxidative stress, and it can thus help prevent neuronal cells from apoptosis [26]. Our previous study revealed SHXT possess neuroprotective effect against activated microglia- and 6-OHDA-induced toxicity [14]. The present results further showed SHXT ameliorated MPP^+^-induced oxidative stress by inhibiting ROS production and gp91^phox^ expression. It also upregulated antioxidant protective systems by increasing both levels of GSH and SOD. Moreover, ROS production contributes to the apoptotic processes found in PD [27]. The release of cytochrome *c* from mitochondria to cytosol could form apoptosome with apoptosis-activating factor (Apaf-1) and procaspase-9, leading to the activation of caspase-9 and caspase-3 [28]. Caspase-3 has been demonstrated to participate in MPP^+^-induced apoptosis and is regulated by mitochondria dysfunction-mediated ROS overproduction [29]. The present results indicated that SHXT not only attenuated MPP^+^-induced oxidative stress, but it also downregulated MPP^+^-induced apoptotic death and molecular events including cytochrome *c* release and caspase cascade activation.

 The most debilitating symptom of PD is the loss of motor control. The MPTP mouse model caused pathophysiology similar to patients with PD [30]. The ability to reflect behavior abnormalities of PD is one of the most useful characteristics of this animal model. According to our results, mice indeed display locomotor abnormalities 7 days after MPTP injection (20 mg/kg, 4 times, 2 h interval). SHXT pretreatment improved locomotor activity in MPTP mice. Moreover, TH is the rate-limiting enzyme in the synthesis of catecholamine neurotransmitters such as dopamine, epinephrine, and norepinephrine. TH activity is progressively decreased following the loss of dopamine neurons in the substantia nigra in the patients with PD [20, 21]. Our results showed that SHXT significantly reduced the loss of TH-positive neurons not only in MPP^+^-treated mesencephalic neurons *in vitro* but also in substantia nigra of MPTP mice *in vivo*. These results suggested that SHXT could protect dopaminergic neurons against MPTP-induced toxicity.

 In conclusion, SHXT provides novel neuroprotective effects, at least in part, via enhancing antioxidative defense, attenuating oxidative stress and decreasing apoptotic death in MPP^+^-treated mesencephalic neuron and in MPTP-mouse model of PD. Therefore, SHXT might be a potentially alternative and complementary medicine used in the treatment of PD.
